# Brain Activity Underlying Muscle Relaxation

**DOI:** 10.3389/fphys.2019.01457

**Published:** 2019-12-03

**Authors:** Kouki Kato, Tobias Vogt, Kazuyuki Kanosue

**Affiliations:** ^1^Physical Education Center, Nanzan University, Nagoya, Japan; ^2^Faculty of Sport Sciences, Waseda University, Tokorozawa, Japan; ^3^Institute of Professional Sport Education and Sport Qualifications, German Sport University Cologne, Cologne, Germany

**Keywords:** inhibition, electromyogram, electroencephalogram, coordination, motor-evoked potential

## Abstract

Fine motor control of not only muscle contraction but also muscle relaxation is required for appropriate movements in both daily life and sports. Movement disorders such as Parkinson’s disease and dystonia are often characterized by deficits of muscle relaxation. Neuroimaging and neurophysiological studies suggest that muscle relaxation is an active process requiring cortical activation, and not just the cessation of contraction. In this article, we review the neural mechanisms of muscle relaxation, primarily utilizing research involving transcranial magnetic stimulation (TMS). Several studies utilizing single-pulse TMS have demonstrated that, during the relaxation phase of a muscle, the excitability of the corticospinal tract controlling that particular muscle is more suppressed than in the resting condition. Other studies, utilizing paired-pulse TMS, have shown that the intracortical inhibition is activated just before muscle relaxation. Moreover, muscle relaxation of one body part suppresses cortical activities controlling other body parts in different limbs. Therefore, the cortical activity might not only be a trigger for muscle relaxation of the target muscles but could also bring about an inhibitory effect on other muscles. This spread of inhibition can hinder the appropriate contraction of muscles involved in multi-limb movements such as those used in sports and the play of musical instruments. This may also be the reason why muscle relaxation is so difficult for beginners, infants, elderly, and the cognitively impaired.

## Introduction

Physical activities in daily life as well as during playing sports or musical instruments require a fine control of not only muscle contraction but also relaxation. Until the 21st century, muscle relaxation was simply regarded as the cessation of contraction because research on motor control had been generally focused on muscle contraction. Since muscle relaxation has been markedly overlooked, the neural mechanisms for muscle relaxation have not been as carefully examined as those for contraction. However, an fMRI study performed a few decades ago revealed that muscle relaxation is an active process requiring a degree of cortical activation similar to or even greater and more widespread than that of muscle contraction (Toma et al., 1999). Thus, to understand motor control, and especially that involved with complex activities, an understanding of the mechanisms of muscle relaxation is just as important as comprehending those involved with muscle contraction.

Compared with the motor control system involved with simple muscle relaxation, control of muscle relaxation during multi-limb coordination is poorly understood. Although numerous studies have demonstrated that muscle contraction of one limb interferes with muscle activity in the other limbs (“remote effect”; Baldissera et al., 1998; Swinnen, 2002; Muraoka et al., 2016; Zheng et al., 2018), the behavioral and neural mechanisms involved in this “remote effect” of muscle relaxation are not well understood.

In relation to relaxation, novice players in sports and music often suffer from inadequate and inappropriate muscle contractions. Thus, an awareness of the mechanisms of relaxation might aid in correcting these problems. The requisite knowledge base is currently unavailable. This information could also be utilized to improve problems of involuntary muscle relaxation that occur in neurological diseases such as stroke and Parkinson’s disease dystonia.

In the present review, we will describe how muscle relaxation is involved in human movement, and characterize the current level of understanding of the underlying neuronal mechanisms. We will focus on knowledge gained by utilizing electrophysiological techniques, mainly the electromyogram (EMG) and transcranial magnetic stimulation (TMS).

## Muscle Relaxation and Human Movement

### Sports and Music

Among athletes and musicians, it is generally acknowledged that adequate and proper muscle relaxation is an absolute necessity for a smooth and efficient performance of movements requiring coordination and quick action. However, this is not easy to accomplish. In a lab setting, Ohtaka and Fujiwara (2018) demonstrated that the control error from the desired target force level was significantly greater for muscle relaxation than for contraction. Furthermore, several studies in practical fields have shown that muscle relaxation is more characteristic and specific than contraction. For example, novice players of sport and music often show unintended contractions of inappropriate muscles and insufficiently strong contractions of necessary muscles (Sakurai and Ohtsuki, 2000; Fujii et al., 2009; Yoshie et al., 2009). For example, novice badminton players showed continuous, unnecessary contractions of the triceps brachii when they swung a racket, whereas skilled players exhibited minimal unnecessary contractions (Sakurai and Ohtsuki, 2000). However, after 6 days of training, the unnecessary contractions decreased in the novice players. Fujii et al. (2009) compared activities in agonist and antagonist muscles during the playing of a drum among novices with no experience, experts, and the world’s fastest drummer. During cyclic bimanual drumming using handheld drumsticks, a relatively large amount of activity in the antagonist muscles together with the activity in the forearm agonist muscles was observed in the novice drummers (i.e., co-contraction). On the other hand, expert drummers were able to suppress co-contraction in the antagonist muscles (i.e., relaxation of unnecessary muscles). The suppression of co-contraction was particularly dramatic in the world’s fastest drummer (Fujii et al., 2009). The neural mechanisms of these differences in muscle activity between novice and expert remain unclear. However, the athletes’ motor cortex does show plastic changes. For example, studies utilizing TMS demonstrate that differences in cortical excitability are evident after a year of experience in athletes, and there is a lower resting motor threshold and higher motor-evoked potential (MEP) elicited by TMS for karate athletes as compared to those of non-athletes (Moscatelli et al., 2016). Furthermore, these changes in the corticospinal tract are reflected in changes in the simple reaction time. Cortical changes were also found for the sport of archery, where proper relaxation of the “pulling hand” is critical. Vogt et al. (2017) utilized an electroencephalogram (EEG) to monitor cortical activity during archery shots, and demonstrated higher activity in the motor area for the skilled novices as compared to less-skilled novices.

Previous studies have evaluated situations in which anxiety hinders appropriate muscle relaxation. Yoshie et al. (2009) set up a competition in which they recorded EMG activities from intermediate pianists. Muscle activities in the biceps brachii and upper trapezius in the competition showed a relative increase compared to those recorded during a rehearsal, and a strong co-contraction in the antagonistic muscles was observed only in the competition. It is quite clear that strong co-contractions of antagonistic muscles produce deficits in physiological efficiency and, among other things, produce muscle fatigue (Lay et al., 2002). Not surprisingly, Yoshie et al. (2009) reported that performance quality was higher in the rehearsal than during the competition.

### Neurological Disorders

Impairment of muscle relaxation (i.e., myotonia) is involved in a wide spectrum of movement disorders such as myotonic dystrophy, dystonia, stroke, and Parkinson’s disease.

#### Myotonic Dystrophy

Myotonic dystrophy (MD) is an inherited disorder, and is the most common form of adult onset muscular dystrophy (Harper et al., 2002; Khoshbakht et al., 2014). MD is characterized by prolonged muscle contractions and an inability to properly relax target muscles after a contraction. For instance, it is difficult for an MD patient to release their hold on a cup or after shaking hands.

Neurophysiological studies of MD patients showed a decrease in corticospinal excitability (Oliveri et al., 1997), disinhibition in the somatosensory cortex (Liepert et al., 2001), and an increase in central motor thresholds (Mochizuki et al., 2001). Movement-related cortical potentials (MRCPs) appear prior to self-initiated voluntary movements for both contraction and relaxation, and reflect movement preparation processing (Rothwell et al., 1998; Shibasaki and Hallett, 2006; Vogt et al., 2017, 2018). MRCPs decreased in patients with MD as compared to patients with other neuromuscular disorders (Mitsuoka et al., 2003). How these changes in neurophysiological parameters are related to the difficulty in relaxation seen in MD patient is still an open question.

#### Dystonia

Dystonia is a syndrome of sustained involuntary muscle contractions in which the patient exhibits frequent twisting, repetitive movements, and abnormal postures (Fahn, 1988). Dystonia is often characterized by a co-contraction of agonist and antagonist muscles. Focal dystonia, the most common form of dystonia, is often task-specific and affects only a single body part. This form of dystonia is termed Focal Task-Specific Dystonia (FTSD) (Pirio Richardson et al., 2017). At some point, the repetitive and precise performance of specific motor actions, such as writing or playing a musical instrument, becomes a trigger for muscle spasms. These contractions interfere with the specific performance, while other actions are unaffected (Jankovic and Ashoori, 2008; Altenmüller et al., 2012; Mohammadi et al., 2012; Quartarone and Hallett, 2013). When patients with dystonia, including FTSD or writer’s cramp (WC), try to move their body parts, the motions are slower and clumsier than expected (Currà et al., 2004). Indeed, EMG bursts are usually prolonged when patients with FTSD or WC carry out simple, rapid movements (van der Kamp et al., 1989; Berardelli et al., 1996). Although the reaction time for simple muscle contraction is similar for FTSD patients and healthy individuals, the reaction time for muscle relaxation is significantly prolonged in the FTSD patients (Buccolieri et al., 2004a,b). Musician’s dystonia is also a type of FTSD that affects the most active parts of the body that are involved with playing such musical instruments as the piano, violin, guitar, flute, clarinet, horn, and tabla. The particular muscles that develop abnormal activation are dependent upon the specific instrument (Stahl and Frucht, 2017; Sadnicka et al., 2018). For example, abnormal involuntary finger flexion is observed in pianists and violinists, while extension of lumbrical muscles of the hand is observed in woodwind and brass players (Conti et al., 2008). The symptoms observed reflect a type of pathological brain plasticity. FTSD is caused by an exaggeration of brain changes that are required to achieve advanced musical skills (Sussman, 2015). Although no current treatment is reliably effective and the disorder generally ends the career of the afflicted musicians, suppression of the debilitating cramps of musician’s dystonia by botulinum injection has been reported (Vecchio et al., 2012).

The slowness of desired movements and the insufficient muscle relaxation seen in these patients might be caused by deficits occurring in both the cortical area and in the spinal cord. The MRCP observed during voluntary muscle relaxation is suppressed in patients with dystonia (Yazawa et al., 1999), suggesting that cortical deactivation, particularly in the inhibitory circuits involved with muscle relaxation, might be the cause of the motor dysfunction (see section “Motor Cortex”). Indeed, studies with paired-pulse TMS demonstrated a reduction in intracortical inhibitions in dystonic patients (Ridding et al., 1995; Chen et al., 1997). Furthermore, recent studies suggest that alterations in activity, connectivity, and structure of the cerebellum are associated with dystonia (Shakkottai et al., 2017; Tewari et al., 2017). For instance, a TMS study revealed that cerebellar modulation of motor cortex excitability was suppressed in patients with focal dystonia (Brighina et al., 2009). However, studies that applied transcranial direct current stimulation (tDCS) to the cerebellum did not show consistent results. That is, while Bradnam et al. (2015) demonstrated that anodal tDCS over the cerebellum induced an improvement of handwriting and circle drawing tasks, found that anodal tDCS applied over the cerebellum failed to improve writing ability for people struggling with writer’s cramp. In the future, non-invasive brain stimulation such as TMS, repetitive TMS, and tDCS on the cerebellum might be a valuable therapeutic tool for enhancing the quality of daily activities in dystonia patients (Lozeron et al., 2016). Dystonia may also result from abnormalities at the spinal level, and is mediated *via* a dysfunction of spinal presynaptic inhibitory mechanisms involving Group I and III afferents (Priori et al., 1995; Lorenzano et al., 2000).

#### Parkinson’s Disease

Parkinson’s disease is a degenerative disorder of the central nervous system. The death of dopaminergic cells in the substantia nigra is the primary cause of the observed motor symptoms. Early in the course of the disease, motor symptoms are the most obvious diagnostic characteristic. These include a resting tremor of body parts, extrapyramidal rigidity, and bradykinesia.

The behavior seen in muscle relaxation tasks changes concurrently with the increased symptom severity that occurs as the motor symptoms of Parkinson’s disease progress. This can be quantified by measuring the time required for relaxation of force from a baseline value to zero. This relaxation time is prolonged in patients with Parkinson’s disease as compared to healthy individuals (Wing, 1988; Jordan et al., 1992). Longer relaxation times are associated with higher bradykinesia scores, even in Parkinson’s disease individuals receiving medication (Grasso et al., 1996). Since presynaptic inhibition in the spinal cord is reduced in patients with Parkinson’s disease, and most markedly on the side with the symptoms. This could be one of the reasons why relaxation time is longer (Lelli et al., 1991) in these patients. Recently, Silva-Batista et al. (2017) reported that instability resistance training (10–12 repetitions of halfsquat, latissimus dorsi pulldown, plantar flexion, chest press, and leg press) decreased relaxation times for Parkinson’s disease patients (Silva-Batista et al., 2017). The authors speculated that greater demands on the central neural systems during the training resulted in improving the descending neural drive, and hence led to a shorter relaxation time.

#### Stroke

Stroke involves a loss of specific brain areas, and their related functions, due to a disturbance in the blood supply to the brain. Strokes in motor areas are quite common, and if a particular hand is involved, the capacity to manipulate objects in that hand is diminished (Parker et al., 1986; Gray et al., 1990; Nakayama et al., 1994). Such deficits are not only involved in muscle contraction (Kamper and Rymer, 2001; Cruz et al., 2005), but also produce a deficiency in relaxation of the paretic limb (Nowak et al., 2003, 2007; Seo et al., 2009). In such patients, Nowak et al. (2003, 2007) documented a delay in the completion of muscle relaxation and an insufficient release of grip force during grip-and-lift tasks. Furthermore, delays in grip initiation (contraction) and termination (relaxation) were considerably longer for the paretic hand (1.9 and 5.0 s) than the nonparetic hand (0.5 and 1.6 s). Corresponding values were even lower for normal subjects (0.2 and 0.4 s) (Seo et al., 2009). The data also indicate that elongation of response time in paretic patients is greater in the relaxation phase than in the contraction phase.

As pointed out above, movement disorder symptoms of neurological patients are observed for both muscle relaxation as well as muscle contraction. Recently, a number of studies have documented a repetitive TMS-induced enhancement of brain cortical excitability and synaptic plasticity in patients with chronic subcortical ischemic vascular disease as well as in those with vascular-related cognitive impairment (Lanza et al., 2007; Pennisi et al., 2015) or vascular-related mood disorders (Cantone et al., 2017). However, further study is required before this technique can be used to improve the dysfunction related to muscle relaxation.

## Neural Mechanisms Underlying Muscle Relaxation

### Motor Cortex

**Figure 1:**
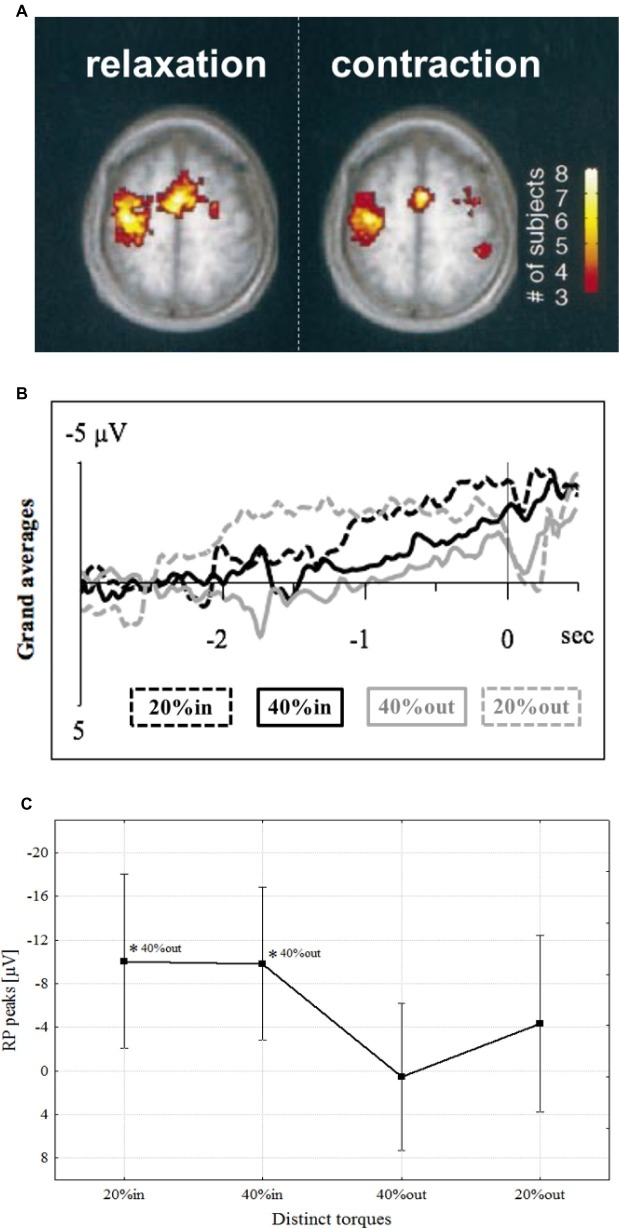
**(A)** Activated areas during muscle relaxation and contraction (Toma et al., 1999). **(B)** Grand average of readiness potential with confidence intervals at distinct torques, i.e., 20% (dashed lines in grand averages) and 40% (continuous lines in grand averages), preceding contraction (in; black in grand averages) or relaxation onsets (out; gray in grand averages) of one continuous motor task sequence over M1 (Vogt et al., 2018). **(C)** Mean readiness potential peaks (μV) at distinct torque values. The levels of significance are marked by asterisks (*p < 0.05), each referenced with annotations respectively (Vogt et al., 2018).

Muscle relaxation began to get attention after a study utilizing functional magnetic resonance imagery (fMRI) revealed that activity in the primary motor cortex (M1) and supplementary motor area (SMA) increased during voluntary muscle relaxation as well as during muscle contraction (Figure 1A; Toma et al., 1999). Corresponding to these brain activities, an MRCP was observed preceding muscle relaxation in the region of the SMA (Terada et al., 1995, 1999; Yazawa et al., 1998; Vogt et al., 2018), pre-SMA (Yazawa et al., 1998), and M1 (Rothwell et al., 1998; Shibasaki and Hallett, 2006; Pope et al., 2007; Vogt et al., 2018). Furthermore, our research group found that an MRCP was observed in M1 that preceded not only simple relaxation, but also sequential relaxation, such as the rapid decline in force from isometric contractions of 40–20% MVC (Figures 1B,C; Vogt et al., 2018). Thus, muscle relaxation, either complete or incomplete, requires a preparatory stage, just as does contraction.

**Figure 2:**
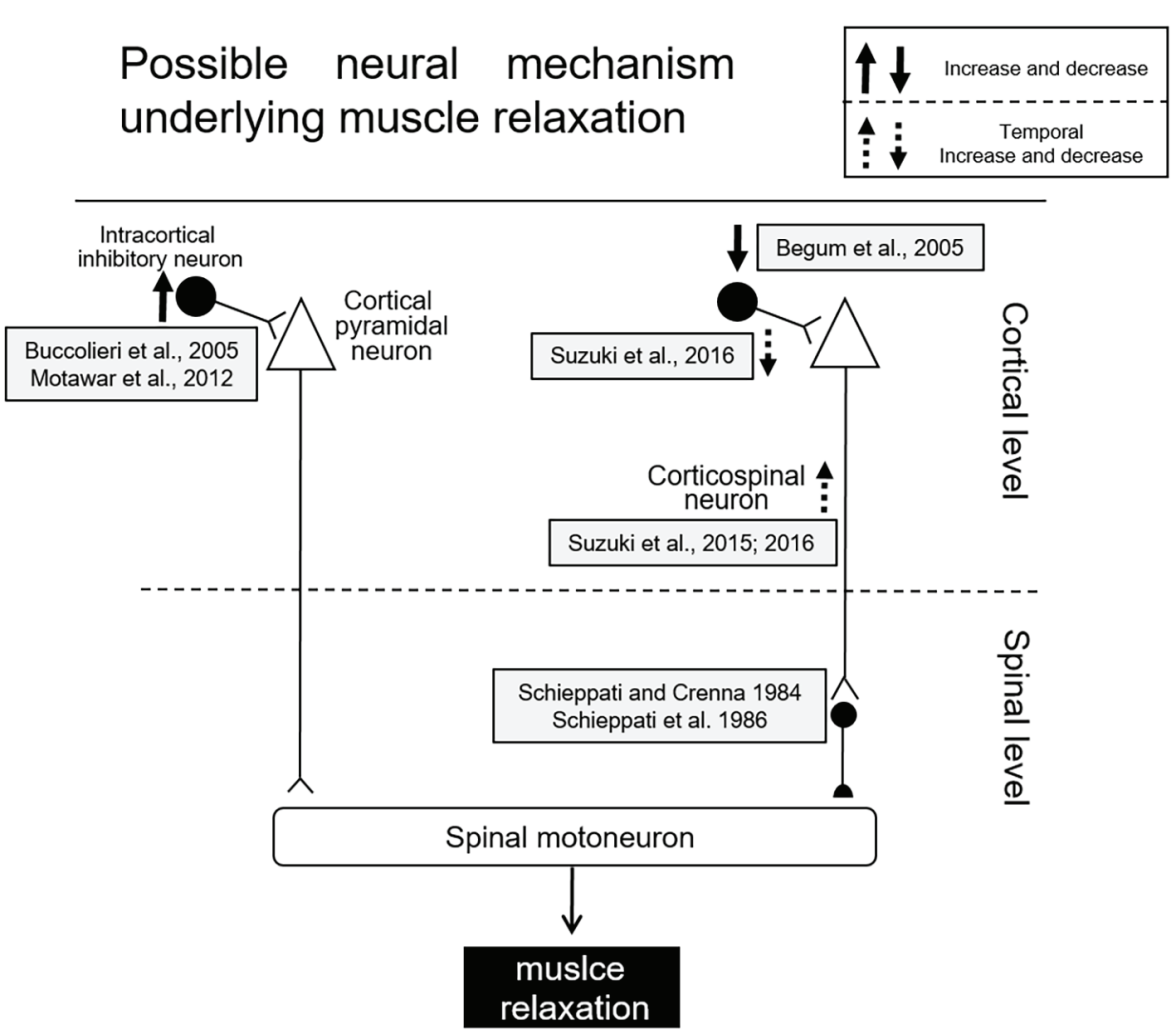
Schematic diagram of possible mechanisms for muscle relaxation (Begum et al., 2005).

Several studies utilizing transcranial magnetic stimulation (TMS) have been performed in order to elucidate the cortical mechanisms of muscle relaxation more in detail. During muscle relaxation of the hand, a decrease in excitability of the corticospinal tract controlling the relaxing of the involved muscles was observed as compared to the resting condition (Buccolieri et al., 2004a,b; Begum et al., 2005; Motawar et al., 2012). Furthermore, the reduction in corticospinal excitability was observed even when a mental representation of muscle relaxation without any overt contraction was involved (i.e., motor imagery) (Kato et al., 2015a,b; Kato and Kanosue, 2018). One possible mechanism for decreasing corticospinal excitability during muscle relaxation could be the activation of intracortical inhibitory circuits (Figure 2). One such circuit involves local GABAergic connections in the motor cortex (short-interval intracortical inhibition: SICI). The mechanism of SICI was first assessed by Kujirai et al. (1993), utilizing the paired-pulse TMS method. Prior to a test stimulus, a conditioning stimulus is given to the same M1 area as was the test stimulus. The intensity of the conditioning stimulus was set below the resting motor threshold, and the intensity of the suprathreshold test stimulus was usually adjusted so as to elicit MEPs with a peak-to-peak amplitude of 1 mV. When these two stimuli were delivered with interstimulus intervals of 1–5 ms, the MEP elicited with the test stimulus became smaller than that elicited by a single test stimulus. The reduction of the test MEP was considered to reflect the inhibition within M1. Reynolds and Ashby (1999) showed that the SICI decreases just prior to the onset of a contraction, and hence cortical excitability is increased in the M1 area innervating the target muscle. To the contrary, the SICI increased prior to muscle relaxation onset (i.e., offset of EMG activity), at a time corresponding to decreased cortical excitability in the target muscle (Buccolieri et al., 2004a,b). Furthermore, Motawar et al. (2012) indicated that muscle relaxation accompanies an enhancement of SICI, after which there is an induced gradual increase of SICI with a progression of the relaxation phase just before relaxation onset. On the other hand, Begum et al. (2005) found that SICI decreased prior to relaxation onset (thus increasing disinhibition), and postulated the activation of spinal inhibitory interneurons due to a decrease in SICI might augment relaxation of the target muscles (Begum et al., 2005).

Another previous study, utilizing the H-reflex technique, has shown muscle relaxation may be mediated by the corticospinal activation of spinal inhibitory presynaptic interneurons (Schieppati and Crenna, 1984; Schieppati et al., 1986). Recently, Suzuki et al. (2015, 2016) have demonstrated that corticospinal excitability is temporally enhanced only in the period from 80 to 60 ms before relaxation onset and the SICI is temporally reduced during that period. Therefore, an SICI during muscle relaxation might rapidly change depending upon the stage (i.e., time course) of relaxation. Furthermore, Suzuki et al. (2015) proposed that the temporal facilitation of motor cortex excitability induced facilitation of spinal inhibitory interneurons; this might be a trigger necessary for the termination of muscle contraction (Sugawara et al., 2016). Although the mechanisms involved in muscle relaxation are still being investigated, activation of both intracortical and spinal inhibitory processes is likely involved in muscle relaxation. Schematic diagrams for the mechanism of muscle relaxation are displayed in Figure 2.

### Brain Regions Other Than Motor Cortex

In addition to M1 and SMA, regions such as the dorsolateral prefrontal cortex (DLPFC), anterior cingulate cortex (ACC), basal ganglia, and cerebellum might well be involved in muscle relaxation. Spraker et al. (2009) found greater activity in the ipsilateral right DLPFC and the ACC during gradual generation and relaxation of the right hand grip force. DLPFC is related to the inhibition of an anticipated motor task such as No-go trials of the Go/No-go task, which is widely utilized to investigate the inhibitory processes involved in motor control (Waldvogel et al., 2000; Nakata et al., 2006). While relaxation involves the termination of a contraction that has already occurred, the No-go trial involves canceling a contraction that is about to be executed. One of corticobasal ganglia loops, the “hyper-direct pathway,” conveys cortical inputs to the substantia nigra pars reticulata through the subthalamic nucleus (STN). This pathway is thought to be related to motor programs of inhibition such as those involving the No-go trial and other types of relaxation. Event-related fMRI studies have demonstrated that the STN is strongly involved in the inhibitory process during No-go trials (Aron and Poldrack, 2006; Aron et al., 2007). The relationship between relaxation and the cerebellum remains poorly understood. Tanaka et al. (2018) did report that a disturbance to the cerebellum with TMS produced no changes in the “imagery” of muscle relaxation. Thus, the cerebellum might not be involved in actual muscle relaxation.

The above studies investigated the neural mechanisms involved with muscle relaxation, of simple movements and/or a single muscle. However, in order to perform most movements in daily life as well as in sports, simultaneous control of both muscle contraction and muscle relaxation in multiple muscles is essential.

## Coordination of Multi-Limb Muscles Underlying Relaxation

In order to perform various movements in daily life as well as in sports, simultaneous control of many muscles in multiple limbs is necessary. The coordination of multi-limb movement involving both contraction and relaxation is quite complicated and is known to be “not just a simple addition of activities of muscle in different limbs and the other’s activity” (Swinnen, 2002). For example, when performing repetitive cyclic movements of both hands or ipsilateral limbs, the movements interfere with each other (Remote effect; Kelso et al., 1979; Nakagawa et al., 2015; Muraoka et al., 2016). During the cyclic movement of ankle dorsiflexion and planterflexion, the corticospinal excitability of resting ipsilateral muscles in the forearm (flexor and extensor muscles) changes depending on the phase of ankle movement. That is, corticospinal excitability of the pronated wrist extensor increases in the dorsiflexion phase of the ankle movement, while that of the flexor increases in the planterflexion phase (Borroni et al., 2004). This remote effect can also be demonstrated with isometric contraction, and this effect is intensified with increasing force levels (Tazoe et al., 2007).

**Figure 3:**
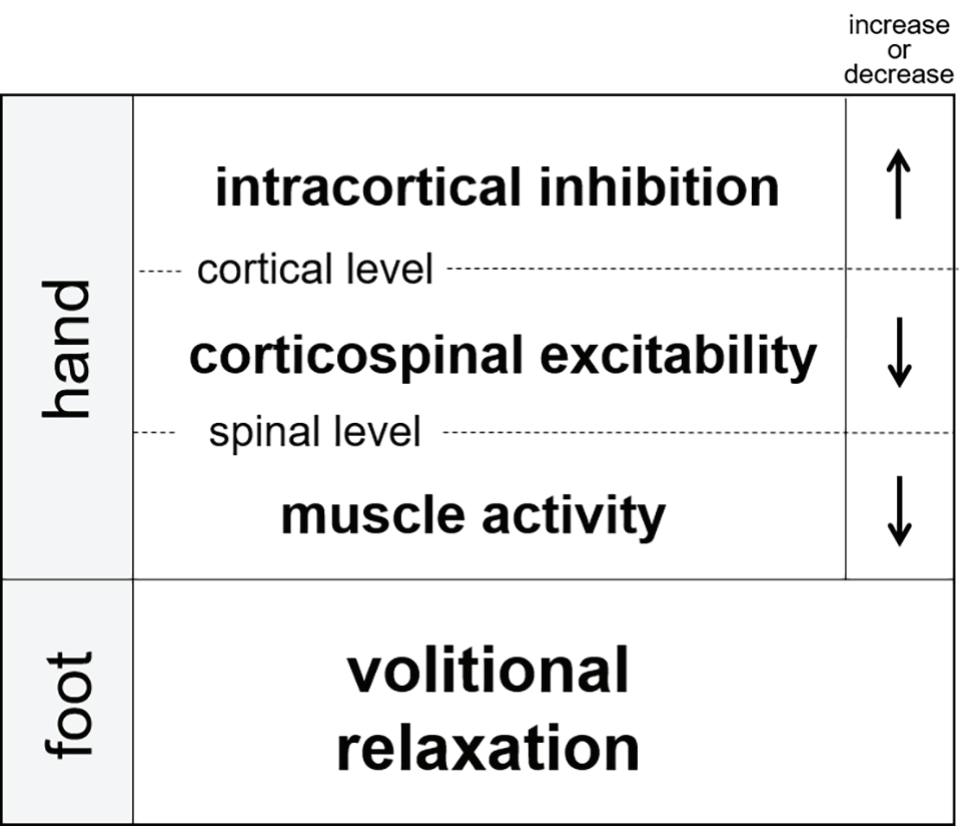
Changes in intracortical inhinhibition, corticospinal excitability, and muscle activity of the hand during volitional relaxation of the foot (Kato et al., 2017).

Since muscle relaxation is an active process requiring cortical activation (Toma et al., 1999), relaxation might be also have remote effects as has been observed for contraction. Recently, our research group demonstrated that muscle relaxation in one limb suppressed muscle activity in the other ipsilateral limb (Figure 3; Kato et al., 2014, 2015a,b). In these experiments, the participants were instructed to execute a simultaneous relaxation and contraction of the ipsilateral hand and foot. Although the subjects tried to separately relax and contract their hand and foot, the EMG activity of contracted muscle in one limb became weakened when it was executed simultaneously with relaxation in the other limb (as compared with the contraction made alone). Therefore, muscle relaxation in one limb suppresses muscle activity of the other (ipsilateral) limb. This is the opposite to the effect of contraction. Next, in order to clarify the neural mechanisms underlying the suppression, TMS to the contralateral forearm region of the primary motor cortex was applied at various timings during and after muscle relaxation of tibialis anterior (the end of ankle dorsiflexion). As a result, corticospinal excitability of the pronated forearm extensor was temporally suppressed during relaxation of the ankle dorsiflexor. Likewise, excitability of the forearm flexor was suppressed during the planterflexor’s relaxation (Figure 3, Kato and Kanosue, 2016a; Kato et al., 2016b). Therefore, we suggest that muscle relaxation of the foot dorsiflexor produces a state in the hand muscles such that a required contraction is difficult. We also used paired-pulse TMS to investigate SICI for the forearm muscles during ipsilateral ankle relaxation. The results revealed that SICI in the M1 forearm region increased during relaxation as compared to that of the resting condition in the ankle (Figure 3; Kato et al., 2016b). This increase in SICI was observed even when the amplitude of the test MEP during relaxation was adjusted to the same amplitude level as that in the resting condition. Therefore, muscle relaxation of one muscle induced a temporal increase in SICI for the other limb, and hence, corticospinal excitability was decreased.

During Go/No-go tasks, moreover, the MEP amplitude of a target muscle decreased in response to a single TMS to the M1 after the No-go stimulus, and increased after the Go stimulus (as compared to the resting condition) (Leocani et al., 2000; Waldvogel et al., 2000; Yamanaka et al., 2002; Nakata et al., 2006). Interestingly, the decrease in MEP amplitude during the No-go task was also observed in the antagonist of the target muscle (Hoshiyama et al., 1997). In addition, the reduction in MEP amplitude was also observed for not only antagonistic muscles, but also for the ipsilateral and contralateral homologous muscles in the limbs that were not directly involved (Leocani et al., 2000; Badry et al., 2009). These results, which indicate a widespread suppressive effect, correspond well with the remote inhibitory effects of relaxation, and suggest that the neural mechanisms of No-go and relaxation at least partially overlap.

## Conclusions and Future Study

Muscle relaxation requires a characteristic brain activation similar to that of muscle contraction. In the playing of sports or musical instruments, as well as in the rehabilitation of movement disorders, although appropriate muscle relaxation is essential for desired movements, it can be quite difficult to accomplish in certain situations. Skilled players are able to use the required muscles to exert appropriate force together with a simultaneous relaxation (or minimal contraction) of unnecessary muscles. On the other hand, when beginners or unskilled players try to perform the same complex set of movements, they are often frustrated by the contraction of muscles in body parts that need to remain relaxed. On such occasions, coaches often say “Relax more!” to the players. However, to execute an appropriate relaxation is not as easy as coaches (and others) may think. First, the relaxation of one particular muscle concurrent with the contraction of other muscles is quite difficult, because the neural circuit controlling the “should-be-relaxed” muscle is activated due to muscle contraction in a different limb (Baldissera et al., 2002; Tazoe et al., 2007). (see section “Sports and Music”) Second, when we focus on the inappropriate contraction and try to suppress it, the muscle contraction necessary to accomplish the required movement is also suppressed due to spreading of the inhibitory effects of relaxation (Kato et al., 2014, 2016b; Kato and Kanosue, 2016a).

Our understanding of relaxation, especially its neural mechanisms, is still fragmentary. We need to clarify details about how muscle relaxation operates during actual performances in sports, music and daily life.

## Author Contributions

All authors listed have made a substantial, direct and intellectual contribution to the work, and approved it for publication.

### Conflict of Interest

The authors declare that the research was conducted in the absence of any commercial or financial relationships that could be construed as a potential conflict of interest.
